# Plasma Neurofilament Light Chain Levels Are Elevated in Children and Young Adults With Wolfram Syndrome

**DOI:** 10.3389/fnins.2022.795317

**Published:** 2022-04-12

**Authors:** Sarah A. Eisenstein, Raveena S. Boodram, Courtney L. Sutphen, Heather M. Lugar, Brian A. Gordon, Bess A. Marshall, Fumihiko Urano, Anne M. Fagan, Tamara Hershey

**Affiliations:** ^1^Department of Psychiatry, Washington University School of Medicine, St. Louis, MO, United States; ^2^Mallinckrodt Institute of Radiology, Washington University School of Medicine, St. Louis, MO, United States; ^3^Department of Neurology, Washington University School of Medicine, St. Louis, MO, United States; ^4^Charles F. and Joanne Knight Alzheimer’s Disease Research Center, Washington University School of Medicine, St. Louis, MO, United States; ^5^Department of Pediatrics, Washington University School of Medicine, St. Louis, MO, United States; ^6^Department of Cell Biology, Washington University School of Medicine, St. Louis, MO, United States; ^7^Department of Medicine, Washington University School of Medicine, St. Louis, MO, United States; ^8^Division of Endocrinology, Metabolism, and Lipid Research, Washington University School of Medicine, St. Louis, MO, United States; ^9^Department of Pathology and Immunology, Washington University School of Medicine, St. Louis, MO, United States; ^10^Hope Center for Neurological Disorders, Washington University School of Medicine, St. Louis, MO, United States

**Keywords:** Wolfram syndrome, neurofilament light chain, neurodegeneration, axonal injury, thalamus, *WFS1* gene

## Abstract

Wolfram syndrome is a rare disease caused by pathogenic variants in the *WFS1* gene with progressive neurodegeneration. As an easily accessible biomarker of progression of neurodegeneration has not yet been found, accurate tracking of the neurodegenerative process over time requires assessment by costly and time-consuming clinical measures and brain magnetic resonance imaging (MRI). A blood-based measure of neurodegeneration, neurofilament light chain (NfL), is relatively inexpensive and can be repeatedly measured at remote sites, standardized, and measured in individuals with MRI contraindications. To determine whether NfL levels may be of use in disease monitoring and reflect disease activity in Wolfram syndrome, plasma NfL levels were compared between children and young adults with Wolfram syndrome (*n* = 38) and controls composed of their siblings and parents (*n* = 35) and related to clinical severity and selected brain region volumes within the Wolfram group. NfL levels were higher in the Wolfram group [median (interquartile range) NfL = 11.3 (7.8–13.9) pg/mL] relative to controls [5.6 (4.5–7.4) pg/mL]. Within the Wolfram group, higher NfL levels related to worse visual acuity, color vision and smell identification, smaller brainstem and thalamic volumes, and faster annual rate of decrease in thalamic volume over time. Our findings suggest that plasma NfL levels can be a powerful tool to non-invasively assess underlying neurodegenerative processes in children, adolescents and young adults with Wolfram syndrome.

## Introduction

Wolfram syndrome is an ultrarare genetic disorder, with features including childhood-onset insulin dependent diabetes mellitus, optic nerve atrophy, sensorineural hearing loss, and diabetes insipidus (Wolfram, 1938; Barrett et al., 1995; Minton et al., 2003). Affecting approximately 1/500,000 worldwide, Wolfram syndrome is a devastating disease, with reported shortened lifespan due to health complications (Barrett et al., 1995; Minton et al., 2003). Wolfram syndrome is caused by pathogenic variants in the *WFS1* gene, which encodes wolframin, a transmembrane ER glycoprotein involved in intracellular calcium homeostasis and regulation of unfolded protein response. In Wolfram syndrome, absent or reduced levels of wolframin disrupt normal ER functioning, leading to ER stress-induced apoptosis (Takeda et al., 2001; Ishihara et al., 2004; Fonseca et al., 2005; Riggs et al., 2005; Yamada et al., 2006; Akiyama et al., 2009; Fonseca et al., 2009; Fonseca et al., 2010).

Recent clinical and brain MRI data from our group’s ongoing natural history study of Wolfram syndrome in children, adolescents and young adults describe an early neurophenotype of ophthalmologic deficits, impaired balance, smell identification, and hearing. Brain MRI analyses from our study and case studies of adults with Wolfram syndrome reveal reduced volumes in ventral pons, cerebellar white matter, thalamus, optic nerve, and total ICV (Rando et al., 1992; Saiz et al., 1995; Barrett et al., 1997; Hadidy et al., 2004; Pakdemirli et al., 2005; Yang et al., 2005; Ito et al., 2007; Mathis et al., 2007; Nickl-Jockschat et al., 2008; Waschbisch et al., 2011; Hershey et al., 2012; Pickett et al., 2012a,b; Karzon and Hullar, 2013; Marshall et al., 2013; Hoekel et al., 2014; Bischoff et al., 2015; Lugar et al., 2016; Zmyslowska et al., 2019; Samara et al., 2020), among other regions, even early in the disease progression (Hershey et al., 2012; Lugar et al., 2016, 2019). Over time and age in children, adolescents and young adults, white matter volume increases in controls but specific white matter volumes (brainstem and ventral pons) decrease in Wolfram syndrome (Lugar et al., 2019). In addition, gray matter subcortical thalamic and cerebellar cortex volumes remain stable in controls but decrease over time in Wolfram syndrome (Lugar et al., 2019). Microstructural integrity in major white matter tracts is lower in individuals with Wolfram syndrome relative to controls (Lugar et al., 2016) and declines over time in the brain’s visual pathway accompanied by progressive deficits in visual acuity (Hoekel et al., 2018). These findings suggest that early neurodevelopmental deficits and neurodegenerative processes, accompanied by worsening clinical severity, occur in Wolfram syndrome.

While MRI measures have proven instrumental in improving our understanding of the disease, they are costly, time-consuming, and require specialized on-site equipment and expertise. In contrast to MRI, a biofluid-based measure of neurodegeneration would be less invasive, easily standardized and repeatable, and able to be performed remotely and in individuals with contraindications for MRI. Thus, such a measure would be extremely useful for ongoing and future clinical trials designed to slow or halt neurologic progression in Wolfram syndrome.

One fluid biomarker protein, NfL, has shown excellent disease-monitoring potential in common neurodegenerative diseases. Neurofilaments are components of the microskeleton and are between microfilaments and microtubules in size (Gaetani et al., 2019). They maintain axonal caliber, facilitate the radial growth of axons, and ensure the structural integrity of neurons and their processes (Yuan et al., 2012, 2015, 2017; Yuan and Nixon, 2016; Gaetani et al., 2019). NfL is the most abundant component of axonal scaffolding and is released into CSF and blood during normal aging and following neuroaxonal injury in a range of neurological conditions, including inflammation, trauma, cerebrovascular disease and neurodegeneration (Petzold, 2005; Bacioglu et al., 2016; Zetterberg, 2016; Disanto et al., 2017; Bridel et al., 2019; Gaetani et al., 2019; Khalil et al., 2020). Although initially studied only in the CSF, recent technological improvements in sensitivity have made it possible to measure NfL in the blood. Serum and plasma NfL levels are highly correlated with CSF levels in disease states (Bacioglu et al., 2016; Disanto et al., 2017; Hansson et al., 2017; Piehl et al., 2018; Harp et al., 2019; Preische et al., 2019). Serum and plasma NfL, which are obtained through blood draws rather than more invasive and uncomfortable lumbar punctures necessary for CSF NfL, are measurable in healthy individuals, and appear to remain stable and at low levels from ∼6–18 years of age with a yearly estimated increase of 2.2% in adulthood (Disanto et al., 2017; Harp et al., 2019).

Neurofilament light chain presence in blood or CSF reflects neuroaxonal injury and relates to clinical severity and MRI measures in progressive neurological disease. NfL levels are not associated with a specific disease etiology but instead are sensitive to progressive neurodegeneration and may predict onset or progression across many diseases, such MS in adults and children, Alzheimer’s disease (AD), Huntington disease, amyotrophic lateral sclerosis, and spinocerebellar ataxia (Disanto et al., 2017; Mattsson et al., 2017; Ashton et al., 2019; Bridel et al., 2019; Gaetani et al., 2019; Gordon, 2020; Reinert et al., 2020; Coarelli et al., 2021). In addition, elevated NfL predicts worse cognitive function and smaller brain volume in both AD and frontotemporal dementia (Rohrer et al., 2016; Mattsson et al., 2017), decreased cerebellar and pons volumes in spinocerebellar ataxia (Coarelli et al., 2021), decreased cerebellar gray matter volume in children with chronic kidney disease (van der Plas et al., 2021), reduced white matter integrity in dominantly inherited AD (Schultz et al., 2020), and decreased hippocampal volume, cortical thickness, white matter integrity, and worsening cognition in cognitively unimpaired older adults (Mielke et al., 2019). NfL levels are also useful as a biomarker for monitoring therapeutic response. Decreasing NfL levels in pediatric and adult MS patients have been consistently shown following disease-modifying therapy (Disanto et al., 2017; Sejbaek et al., 2019; Hyun et al., 2020; Reinert et al., 2020). NfL has also demonstrated response to treatment in other disease including slowed rates of change in clinical trials of anti-amyloid therapies in dominantly inherited AD (Salloway et al., 2021).

Given this background, it is reasonable to hypothesize that NfL levels may be elevated in Wolfram syndrome, and that this measure could be useful for disease monitoring. The primary aim of this study was to compare plasma NfL levels between children, adolescents, and young adults with Wolfram syndrome and controls consisting of their parents and siblings. Second, in a subset of individuals with Wolfram syndrome, plasma NfL levels at ∼1.8 years after baseline were measured. We hypothesized that baseline and follow-up NfL levels would be elevated in individuals with Wolfram syndrome relative to controls independent of age and that higher NfL levels would relate to worse clinical severity and smaller regional brain volumes in individuals with Wolfram syndrome.

## Materials and Methods

### Participants

Participants with genetically confirmed Wolfram syndrome diagnosis were recruited *via* self or physician referral to attend the annual Wolfram syndrome Research Clinic at Washington University in St. Louis (WUSTL), MO, United States. Participants with Wolfram syndrome and their unaffected parents or siblings attended the clinic between 2010 and 2017. The study protocol was approved by the Human Research and Protection Office at WUSTL and carried out in accordance with the Declaration of Helsinki. Participants <18 years of age gave informed assent, and their parents or legal guardians gave written informed consent. Participants ≥18 years gave written informed consent.

### Plasma Sample Collection

Participants fasted overnight, and blood was collected into EDTA vacutainer tubes on ice and spun down at 1300 *g* × 10 min. Plasma was aliquoted (100 μL) and frozen at −80°C. In total, 65 blood samples were obtained from the Wolfram group in 2014, 2016, and 2017 and 35 from the control group in 2014. Within Wolfram participants, 27 had plasma NfL measures for two consecutive time points, designated hereafter as time points 1 and 2.

### Neurofilament Light Chain Measurements

Plasma NfL levels were assayed in duplicate per manufacturer instructions using the commercially available NfL immunoassay kit (Quanterix NfL Advantage Kit™, Quanterix Corp., United States) on the automated ultrasensitive Simoa^®^ HD-X Analyzer (Quanterix Corp., United States) platform. Samples were diluted 1:2 prior to loading on to the HD-X to reduce the volume of plasma needed for the assay. QC parameters were described previously (Hendricks et al., 2019). The assay required four kits in total. Effects of year that the samples were collected and of separate kits on NfL levels were assessed. No individual sample had CV > 25% in duplicate assays.

### Clinical Disease Severity Measures

#### Wolfram United Rating Scale

The WURS (Nguyen et al., 2012; Bischoff et al., 2015) was administered by a neurologist. The WURS instrument was developed to assess overall disease severity of Wolfram syndrome sequelae (e.g., vision, hearing, motor, urological, neurological, psychological, and mood problems) and validated in a subset of the participants currently described (Nguyen et al., 2012). The maximum score for the subscale used here to indicate clinical severity, the Physical Activity subscale, is 136, with higher scores indicating greater severity (Nguyen et al., 2012).

#### Visual Acuity

Using Snellen optotypes, best-corrected visual acuity was recorded and transformed into Logarithm of the Minimum Angle of Resolution scaled values, with higher values indicative of worse visual acuity, for each participant with Wolfram syndrome as described in Hoekel et al. (2014). Normal visual acuity is 20/20 (logMAR = 0, no loss of visual acuity). Color vision was assessed using Hardy-Rand-Rittler as described in Hoekel et al. (2014). The normal color vision score (number correct) is 51–52. In a study of a subset (*n* = 18) of the participants currently described, mean (range) vision acuity was 20/60 (20/2000–20/20) and color vision score was 13.2 (0–51), with 89 and 94% of individuals with Wolfram syndrome having subnormal visual acuity and deficits in color vision, respectively (Hoekel et al., 2014).

#### Smell Identification

Smell identification was assessed with the University of Pennsylvania Smell Identification Test (Doty et al., 1984) as described in Alfaro et al. (2020). Briefly, participants were asked to scratch and sniff stimuli with microencapsulated odorants and indicate which of four response alternatives best matched the perceived odor. Higher scores indicate more accurate smell identification. Relative to age-matched healthy controls and individuals with Type 1 diabetes, a sample (*n* = 40) including most of the individuals with Wolfram syndrome in the current study had less accurate smell identification (Alfaro et al., 2020).

### Regional Brain Volumes

Regional brain volumes in each participant with Wolfram syndrome were obtained from MRI scans as described in Lugar et al. (2019). Briefly, individuals with Wolfram syndrome underwent MRI scans on a Siemens 3T Tim Trio at the Center for Clinical Imaging Research at Washington University. The analyses described here include data obtained from T1-weighted Magnetization-Prepared Rapid Gradient-Echo (MPRAGE) sequences. Regional brain volumes were determined using Freesurfer 5.3 (Fischl et al., 2002), averaged between left and right hemispheres and corrected for total ICV by dividing regional brain volume by estimated total intracranial volume (Buckner et al., 2004) and scaling the quotient by 1,400,000 mm^3^, an approximately average eTIV. *A priori* regions of interest were selected for analyses based on previous findings of decreased volume over time in a Wolfram patient study sample (*n* = 29), including most of the individuals with Wolfram syndrome described in the current study, compared to controls including ventral pons, brainstem, cerebellar cortex, and thalamus (Lugar et al., 2019).

### Statistical Analyses

Raw plasma NfL levels were log10-transformed to normalize distributions, which is a standard way of analyzing NfL levels (Mattsson et al., 2017; Zeitlberger et al., 2018; Mielke et al., 2019; Preische et al., 2019; Reinert et al., 2020; Goeral et al., 2021). These and other relevant variables were compared between individuals with Wolfram syndrome and the control group with Student’s between-subjects *t*-tests and between time points 1 and 2 in the Wolfram group with Student’s within-subjects *t*-tests. Gender and ethnicity distributions were compared between control and Wolfram groups with Mann–Whitney *U* tests. A one-way ANCOVA was used to determine whether plasma NfL levels differed between control and Wolfram groups when age was controlled. Correlation of plasma NfL levels with age was evaluated within the Wolfram group and within controls using separate Pearson’s *r* analyses. Effects of kit number and plasma sample collection year on plasma NfL levels were assessed with one-way ANOVA. For the group comparisons and correlations with age, alpha was set to *p* < 0.05, as these were primary *a priori* hypotheses. Additional analyses were considered exploratory in the interest of generating testable hypotheses in future studies and so were not corrected for multiple comparisons.

While plasma was collected annually for up to two consecutive time points (time points 1 and 2) in individuals with Wolfram syndrome, clinical and MRI measures were obtained annually for up to 7 years depending on when the participant started attending the clinic. In exploratory analyses within the Wolfram group, we performed Pearson’s *r* or Spearman’s ρ correlations between log10 plasma NfL levels and disease severity and MRI variables obtained during the corresponding clinic year. Annual percent change in volume was calculated for brain regions in which volume related to NfL levels at both time points (thalamus). Specifically, for each participant, average annual percent change in thalamic volume was calculated with the following formula:

$$\frac{\left(\frac{\sum \left(x-\bar{x}\right)\,\left(y-\bar{y}\right)}{\sum {\left(x-\bar{x}\right)}^{2}}\right)}{\bar{y}}\times 100$$

where slope is $\frac{\sum \left(x-\bar{x}\right)\,\left(y-\bar{y}\right)}{\sum {\left(x-\bar{x}\right)}^{2}}$, *x* is age at MRI visit, $\bar{x}$ is mean age across MRI visits, *y* is thalamic volume at MRI visit and $\bar{y}$ is mean thalamic volume across MRI visits. The mean thalamic volume across MRI visits was used to normalize slopes so that variability in thalamic volume over the study period, rather than just at baseline, could be removed from the slope calculation for each participant. Number of MRI visits varied from 2 to 7 in individuals with Wolfram syndrome depending on how many annual clinics were attended.

## Results

### Participants

Descriptive statistics for the control and Wolfram groups are shown in Table 1. Within controls, all had a single time point at which NfL was measured; 28 were parents and 7 were siblings of individuals with Wolfram syndrome. Within the Wolfram group, 38 participants had plasma NfL data from time point 1 and 27 of these individuals also had plasma NfL data from time point 2. Scaled parent education levels were derived from the Barratt Simplified Measure of Social Status (Barratt, 2006) and were averaged when data from both parents were available. For parents, their own scaled education level was used. One individual with Wolfram syndrome had raw plasma NfL levels at both time points 1 (37.8 pg/mL) and 2 (34.2 pg/mL) >3 SD above the respective means, but were < 3SD above log10 means at time points 1 (log10 plasma NfL = 1.58) and 2 (1.53) within the Wolfram group and when controls were included in the calculation. Therefore, this participant’s data points were included in data analyses. Due to many parents (*n* = 28, 80%) in the control group, the Wolfram sample was younger (*t*_71_ = –8.5, *p* < 0.001). Wolfram and control groups did not differ in gender or ethnicity distributions (*p* ≥ 0.63) or in scaled parent education level (*p* = 0.33).

**TABLE 1:** Control and Wolfram group demographics and plasma neurofilament light chain levels.

	All controls	Parents	Siblings	Wolfram	Wolfram with two time points	
*N* (number of participants with NfL samples)	35	28	7	38	27*^a^*	
Female/male	22/13	20/8	2/5	22/16	18/9	
Race (ethnicity)	35 W (12 H)	28 W (10 H)	7 W (2 H)	38 W (11 H)	27 W (9 H)	
Mean scaled parental education ± SD	15.5 ± 4.5*^b^*	15.3 ± 4.6*^b^*	16.3 ± 4.2	14.5 ± 4.2*^c^*	14.9 ± 4.8*^c^*	
					**TP 1** *^d^*	**TP 2**
Median age (years, IQR)	41.0 (35.1–47.9)	44.8 (39.6–49.5)	12.5 (10.3–14.1)	14.4 (5.1–29.7)	14.0 (10.8–20.1)	16.0 (12.8–22.1)
Median plasma NfL (pg/mL, IQR)	5.6 (4.5–7.4)	5.9 (5.0–7.4)	4.5 (2.8–6.4)	11.3 (7.8–13.9)	10.6 (7.2–14.5)	10.7 (8.4–13.8)
Median log10 plasma NfL (IQR)	0.7 (0.7–0.9)	0.8 (0.7–0.9)	0.7 (0.5–0.8)	1.1 (0.9–1.1)	1.0 (0.9–1.2)	1.0 (0.9–1.1)

*NfL, neurofilament light chain; W, White; H, Hispanic; IQR, interquartile range; TP, time point.*

*^a^Of 38 individuals in the Wolfram group, 27 also had data from a second time point.*

*^b^Data missing from two participants.*

*^c^Data missing from four participants.*

*^d^TP 1 and TP 2 columns include data for 27 out of 38 individuals in Wolfram group who had NfL data from both time points.*

### Clinical Disease Severity Variables

Individuals with Wolfram syndrome had worse visual acuity and smaller ventral pons, brainstem, cerebellar cortex and thalamic volumes at time point 2 relative to time point 1 (Table 2) as in previous publications that included a subset of these participants (Hoekel et al., 2014; Lugar et al., 2019). Mean (SD) annual decrease in thalamic volume over all MRI time points was –66.1 (80.9) mm^3^ and thalamic-volume corrected mean (SD) annual decrease in thalamic volume was –1.0% (1.3).

**TABLE 2:** Clinical severity measures in individuals with Wolfram syndrome.

	TP 1	*N* with 1 TP	TP 1*^a^*	TP 2	*N* with 2 TPs
Disease duration (years)	4.5 ± 4.4	37	3.9 ± 4.1	5.6 ± 4.0	26
Plasma glucose (mg/dL)	187.8 ± 68.4	36	187.7 ± 72.5	181.6 ± 84.4	26
HbA1c	7.5 ± 1.5	36	7.5 ± 1.7	7.3 ± 1.1	26
WURS Physical Activity subscale score	5.0 ± 5.7	34	4.3 ± 3.2	4.5 ± 3.7	24
UPSIT total score (number correct)	24.9 ± 7.5	37	24.9 ± 7.8	24.0 ± 7.3	27
Visual acuity (logMAR)	0.56 ± 0.45	34	0.50 ± 0.34	0.56 ± 0.39*	27
Color vision (number correct)	9.2 ± 9.3	30	10.7 ± 9.4	10.2 ± 10	20
Ventral pons volume (mm^3^)	6280 ± 1160	27	6133 ± 1190	6030 ± 1249*	20
Brainstem volume (mm^3^)	15079 ± 1590	18	15079 ± 1590	14879 ± 1649**	18
Cerebellar cortex volume (mm^3^)	46109 ± 4440	26	45573 ± 4393	44712 ± 4420**	19
Thalamus volume (mm^3^)	6530 ± 478	26	6546 ± 381	6429 ± 425**	19
Follow-up duration (years, range)	NA	NA	NA	1.8 (0.99–2.0)	27

*Mean (SD) shown except where noted.*

*TP, time point; NA, not applicable; WURS, Wolfram United Rating Scale; UPSIT, University of Pennsylvania Smell Identification Test.*

**, *p < 0.01, 0.05 relative to time point 1.

*^a^Column includes individuals with Wolfram syndrome who had data from both time points.*

### Quality Control for Neurofilament Light Chain Measures

The average (SD) CV across 72 replicate samples on separate assay plates run by two separate technicians was 5.7% (4.8). For one individual with Wolfram syndrome, a replicate was not analyzed due to a processing error. Given the consistently low CV (i.e., high reproducibility), the single value for this participant was included in analyses. Within 73 time point 1 and 27 time point 2 samples, neither year of sample collection (1-way ANOVA, *p* ≥ 0.36) nor kit number (1-way ANOVA, *p* ≥ 0.27) affected NfL plasma concentrations.

### Plasma Neurofilament Light Chain Level Comparisons Between Control and Wolfram Groups

**FIGURE 1:**
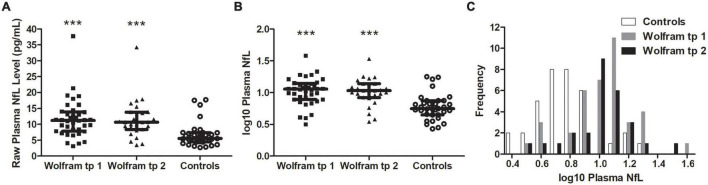
Raw **(A)** and log10 **(B)** plasma NfL levels were higher in the Wolfram group at time points 1 and 2 relative to controls. Results were similar when outlier data were excluded. Median and IQR shown. **(C)** Frequency distribution of log10 plasma NfL levels for control and Wolfram groups at time points 1 and 2. NfL, neurofilament light; tp, time point. ***p < 0.001 relative to controls.

**FIGURE 2:**
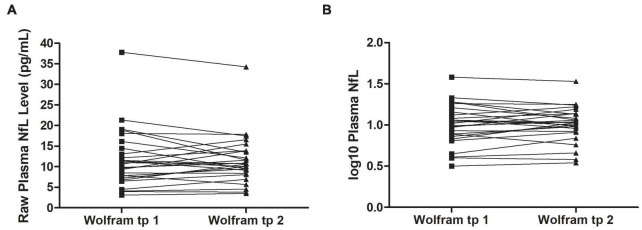
Raw **(A)** and log10 **(B)** plasma NfL levels were similar at time points 1 and 2 within individuals in the Wolfram group who had NfL measures at both time points 1.8 years apart on average (*n* = 27). NfL, neurofilament light; tp, time point.

Raw and log10 plasma NfL levels on average were higher in individuals with Wolfram syndrome at time point 1 [Raw: *t*_(1,71)_ = 4.2, *p* < 0.001, Cohen’s *d* = 1.0; Log10: *t*_(1,71)_ = 5.0, *p* < 0.001, Cohen’s *d* = 1.2] and time point 2 [Raw: *t*_(1,60)_ = 3.9, *p* < 0.001, Cohen’s *d* = 1.0; Log10: *t*_(1,60)_ = 4.5, *p* < 0.001, Cohen’s *d* = 1.1] compared to controls (Table 1 and Figures 1A–C), including when the raw plasma NfL outlier was excluded from these analyses (both time points *p* < 0.001 relative to controls). Raw and log10 plasma NfL levels were higher in individuals with Wolfram syndrome relative to both control group subsets (parents: both time points *p* < 0.001; siblings: both time points *p* ≤ 0.02) including when the Wolfram group outlier was excluded (*p* ≤ 0.01). Neither raw nor log10 plasma NfL levels were different between time points within the Wolfram group (*p* ≥ 0.66) (Table 1 and Figures 2A,B).

### Plasma Neurofilament Light Chain Levels and Age

Both raw and log10 NfL levels were higher in the Wolfram group compared to controls when age was covaried [time point 1: *F*_(1,70)_ = 26.7, *p* < 0.001, Cohen’s *d* = 1.23; time point 2: *F*_(1,59)_ = 24.2, *p* < 0.001, Cohen’s *d* = 1.28], indicating that disease status, not age, drives the difference in NfL levels between groups. Age did not relate to raw or log10 plasma NfL levels within individuals with Wolfram syndrome (Table 3) or within controls (*r*_35_ ≤ 0.31, *p* ≥ 0.07). Results were similar when the raw plasma NfL outlier data point was excluded from the Wolfram syndrome group (*r*_37_ ≤ 0.09, *p* ≥ 0.60).

**TABLE 3:** Correlations between log10 plasma neurofilament light levels with age and measures of clinical severity at time points 1 and 2 in individuals with Wolfram syndrome.

	NfL TP 1 vs. Clinical TP 1	*N*	NfL TP 1 vs. Clinical TP 2	*N*	NfL TP 2 vs. Clinical TP 2	*N*
Age	*r* = 0.21, *p* = 0.21	38	*r* = 0.23, *p* = 0.25	27	*r* = 0.30, *p* = 0.13	27
WURS Physical Activity subscore total	ρ = –0.14, *p* = 0.40	34	*ρ* = –0.07, *p* = 0.72	26	ρ = 0.10, *p* = 0.63	26
UPSIT (number correct)	*r* = –0.26, *p* = 0.12	37	*r* = –0.38, *p* = 0.05*	27	*r* = –0.46, *p* = 0.02*	27
Visual acuity (logMAR)	*r* = 0.34, *p* = 0.05*	34	*r* = 0.40, *p* = 0.04*	27	*r* = –0.46, *p* = 0.02*	27
Color vision (number correct)	*r* = –0.40, *p* = 0.03*	30	*r* = –0.42, *p* = 0.07	20	*r* = –0.59, *p* = 0.01**	20
Ventral pons volume	*r* = –0.24, *p* = 0.23	27	*r* = –0.34, *p* = 0.14	21	*r* = –0.35, *p* = 0.12	21
Brainstem volume	*r* = –0.48, *p* = 0.04*	18	*r* = –0.50, *p* = 0.04*	18	*r* = –0.43, *p* = 0.08	18
Cerebellar cortex volume	*r* = 0.12, *p* = 0.55	26	*r* = 0.21, *p* = 0.37	20	*r* = 0.25, *p* = 0.29	20
Thalamic volume	*r* = –0.36, *p* = 0.07	26	*r* = –0.60, *p* = 0.01**	20	*r* = –0.57, *p* = 0.01**	20
Average annual rate of change in thalamic volume	NA		*r* = –0.52, *p* = 0.01**	24	NA	

NfL, neurofilament light chain; TP, time point; WURS, Wolfram Unified Rating Scale; UPSIT, University of Pennsylvania Smell Identification Test; NA, not applicable. *, **p ≤ 0.05, 0.01. Bold p-values indicate statistical significance at α = 0.05. N = number of participants with data points included in the analysis in the preceding column.

### Plasma Neurofilament Light Chain Levels, Clinical Severity and Neurodegeneration in Individuals With Wolfram Syndrome

**FIGURE 3:**
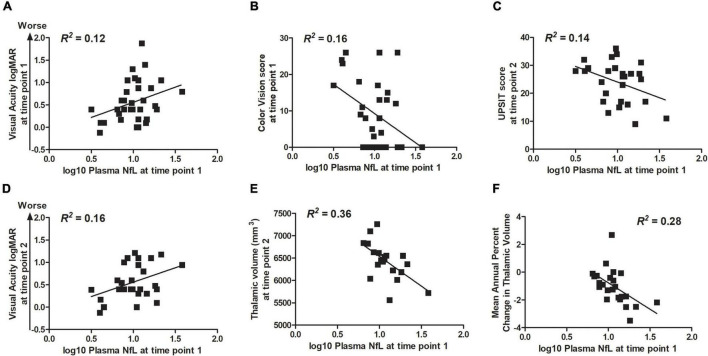
Within the Wolfram group, higher log10 plasma NfL levels at time point 1 related to **(A,B)** worse visual acuity and color vision at time point 1; **(C–E)** worse visual acuity and color vision (data not shown), less accurate smell identification, and smaller thalamic volume at time point 2; and **(F)** faster rate of annual decrease in thalamic volume.

**FIGURE 4:**
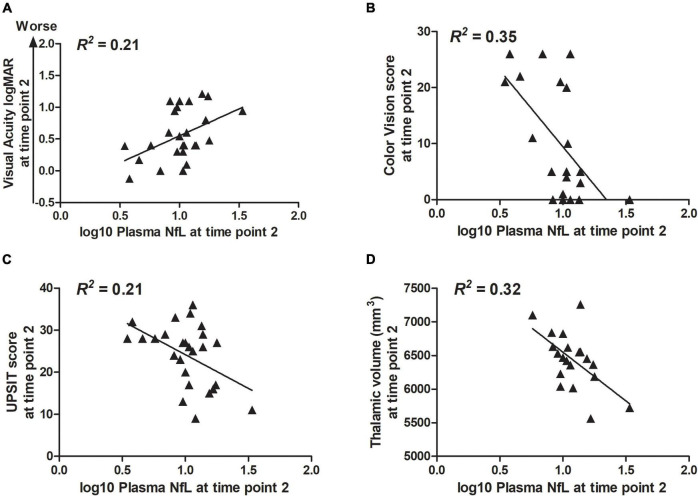
Within the Wolfram group, higher log10 plasma NfL levels at time point 2 related to **(A–D)** worse visual acuity and color vision, less accurate smell identification, and smaller thalamic volume at time point 2.

Correlation statistics for the relationship between log10 plasma NfL levels at time points 1 and 2 with measures of clinical severity are shown in Table 3. Briefly, higher log10 plasma NfL levels at time point 1 related to worse visual acuity and color vision at time point 1, worse visual acuity, less accurate smell identification and smaller brainstem and thalamic volumes at time point 2, and faster annual rate of decrease in thalamic volume [mean (SD) number of annual MRIs = 4.5 (1.6) per participant] (Figures 3A–F). Higher log10 plasma NfL levels at time point 2 related to worse visual acuity and color vision, less accurate smell identification, and smaller thalamic volume at time point 2 (Figures 4A–D).

## Discussion

Similar to other neurological diseases in which neuroaxonal injury is a core feature (Khalil et al., 2018; Gaetani et al., 2019), we found that NfL levels are higher in individuals with Wolfram syndrome compared to controls and related to measures of greater clinical severity and neurodegeneration. Between two time points ∼1.8 years apart, plasma NfL levels did not differ, indicating that any change in plasma NfL levels is not detectable over this brief interval in Wolfram syndrome. Our findings demonstrate that NfL levels are sensitive to clinical presentation and brain health in Wolfram syndrome, indicating that this blood-based marker may have prognostic value and is a promising biomarker to monitor response in future theraputic trials.

For NfL to be of use in detecting clinically relevant severity or rate of neuroaxonal injury, NfL levels must be different in individuals with disease relative to controls. Our primary finding is that plasma NfL levels discriminate between individuals with Wolfram syndrome and controls composed of parents and siblings of individuals in the Wolfram group. In addition, unlike in controls, these levels were higher relative to the normal reference range [median (IQR) plasma NfL = 5.9 (4.3–7.9) pg/mL] established by a previous study of NfL levels in healthy donors aged 24–64 years using the same assay on the SIMOA platform (Harp et al., 2019). The observed plasma NfL levels in individuals with Wolfram syndrome overlap with similar-aged, untreated children with pediatric MS (Reinert et al., 2020) and appear similar to those in people with asymptomatic spinocerebellar ataxia type 3 [median (IQR) NfL = 12.2 (10.2–13.9) pg/mL] (Peng et al., 2020). The latter disease, like Wolfram syndrome, is caused by a genetic mutation with variable expressivity, rate of progression, age of onset, and phenotype, and is accompanied by cerebellar and brainstem atrophy (Brooker et al., 2021). The plasma NfL levels observed here in Wolfram syndrome also overlap with those obtained from older (mean age = 57.4 years) individuals with type 1 diabetes [mean (SD) plasma NfL = 13.3 (6.7) pg/mL]. It is possible that between-group factors other than central axonal injury such as peripheral nerve damage, impaired renal function and/or vascular neuropathy may be responsible for elevated plasma NfL levels in individuals with Wolfram syndrome (Bischof et al., 2018; Khalil et al., 2018; Sandelius et al., 2018; Akamine et al., 2020; Frempong et al., 2021). Future studies using CSF NfL samples and MR diffusion studies of tissue microstructural integrity, known to be impaired in Wolfram syndrome (Lugar et al., 2016), will help to determine whether the elevated NfL is primarily due to central axonal injury or other aspects of Wolfram syndrome (e.g., diabetes, ER dysfunction) (Piehl et al., 2018; Akamine et al., 2020; van der Plas et al., 2021). Unfortunately, the current study was not powered to detect relationships between diffusion MRI-based tissue microstructural integrity and NfL levels since these measures were not always obtained during the same clinic year in individuals with Wolfram syndrome. Nonetheless, elevated plasma NfL levels in individuals with Wolfram syndrome cross-sectionally related to smaller brainstem and thalamic volumes and faster rate of decreasing thalamic volume over time, providing indirect evidence that NfL levels likely reflect the current degree or rate of central axonal injury.

To determine whether NfL levels are altered over time and/or predict future disease activity, longitudinal studies are required over greater than 2 years. In contrast, this interval was sufficient for detection of increased clinical severity and neurodegeneration. Interestingly, in sera from a small sample of teens and young adults with Wolfram syndrome, expression of multiple microRNAs are altered after 2 years follow-up and relate to simultaneous MRI indicators of neurodegeneration including reduced macular average thickness and brainstem volume (Zmyslowska et al., 2020). These preliminary observations suggest that serum microRNA expression, influenced by ER stress, may be a sensitive marker of short-term progression in neurodegeneration in Wolfram syndrome (Zmyslowska et al., 2020). In the current study, NfL levels at time point 1 did relate to worsening accuracy in smell identification and visual acuity and decreased thalamic and brainstem volumes at time point 2, indicating that NfL levels may predict future disease activity in Wolfram syndrome. Of note, our findings are similar in nature to those of a study of spinocerebellar ataxia, in which NfL levels did not change over 24 mos but predicted worsening in clinical severity and decreased cerebellar and pons volumes over this time period (Coarelli et al., 2021). To test whether NfL levels truly predict future disease activity in Wolfram syndrome, a prospective, longitudinal study with sufficient sample size, duration, and sampling frequency is required.

Neurofilament light chain levels fluctuate non-linearly over the lifespan in healthy individuals and, in normal aging, elevated NfL levels cross-sectionally and longitudinally relate to brain volume loss presumably due to increasing levels of neuroaxonal injury (Bridel et al., 2019; Khalil et al., 2020; Ashton et al., 2021). Risk for or presence of disease often alters the relationship between age and NfL levels (Bridel et al., 2019; Khalil et al., 2020; Ashton et al., 2021; van der Plas et al., 2021). The subset of siblings in our control group, composed of children and adolescents, had plasma NfL levels similar to those of the control parents in our study and to serum NfL levels in similarly aged healthy controls in a pediatric MS study (Reinert et al., 2020). The subset of parents in our control group (age range = 26.6–59.7 years) had plasma NfL levels in line with the established reference range for the Quanterix/SIMOA platform (Harp et al., 2019) but tended to skew lower than other published control plasma and serum NfL samples from similarly aged adults (Kuhle et al., 2015; Weydt et al., 2016; Weston et al., 2017; Benatar et al., 2018; Korley et al., 2018; Thompson et al., 2018; Zeitlberger et al., 2018; Clay et al., 2020; Coarelli et al., 2021), likely due to differences in assay methods, specimen type, and variability in age ranges were studied. While, we did not find evidence of a relationship between age and plasma NfL levels at either time point in the Wolfram group, we observed that, in controls, older age related to higher log10 plasma NfL levels (age range = 3.0–59.7 years), albeit at trend-level statistical significance, similar to previous studies that included similarly aged controls (Clay et al., 2020; Hayer et al., 2020). Given the small sample size and limited age range in the Wolfram group (5.1–30.7 years), it is difficult to know whether Wolfram syndrome alters the relationship between age and NfL levels. Future longitudinal studies including a wide age range of individuals with Wolfram syndrome will help determine at what age(s) NfL levels differ from those of controls and if and how they relate to age.

A strength of the current study is the direct comparison of NfL levels between controls and individuals with Wolfram syndrome using the same assays at the same study site. Relatives of individuals with Wolfram syndrome served as convenient case controls that guarded against environmental confounds. Future sampling of unrelated control groups using the same assays at the same study site may yield further insight into differences in NfL levels between individuals with Wolfram syndrome and the general population. There are several weaknesses in the currently described study. The study sample is small due to the rarity of Wolfram syndrome and people with Wolfram syndrome with severe physical and/or psychological impairment that restricted travel to participate are not represented. Finally, the relatively short time between baseline and follow-up blood draws (2 years) limited our ability to detect whether NfL levels change over time in individuals with Wolfram syndrome.

## Conclusion

We show that plasma NfL levels are higher in individuals with Wolfram syndrome relative to parent and sibling controls and that higher NfL levels are related to worse clinical symptoms, smaller brainstem and thalamic volumes, and greater annual percent loss of thalamic volume. Serial NfL measures in Wolfram syndrome using prospective, large, and longitudinal studies are needed to determine whether NfL levels change over time, are prognostic of future disease activity, and reflective of response to treatment in this disease. This study suggests that such an investigation is warranted and could improve future clinical trials for Wolfram syndrome by providing a potential easily obtained outcome measure of neurodegeneration (Khalil et al., 2018).

## Data Availability Statement

The raw data supporting the conclusions of this article will be made available by the authors, without undue reservation.

## Ethics Statement

The studies involving human participants were reviewed and approved by the Human Research and Protection Office at Washington University in St. Louis, MO, United States, and carried out in accordance with the Declaration of Helsinki. Participants < 18 years of age gave informed assent, and their parents or legal guardians gave written informed consent. Participants ≥ 18 years gave written informed consent.

## Author Contributions

SE: formal analysis, writing–original draft preparation, writing–review and editing, and visualization. RB: writing–original draft preparation, formal analysis, and writing–review and editing. CS: validation, investigation, and writing–review and editing. HL: formal analysis, investigation, data curation, and writing–review and editing. BG: writing–review and editing. BM and FU: investigation and writing–review and editing. AF: methodology, resources, writing–review and editing, and supervision. TH: conceptualization, methodology, formal analysis, investigation, resources, writing–original draft preparation, writing–review and editing, supervision, project administration, and funding acquisition. All authors made substantial contributions to the conception or design of the work or the acquisition, analysis, or interpretation of data for the work; drafted the work or critically revised it for important intellectual content; and agreed to be accountable for all aspects of the work.

## Conflict of Interest

FU is a Founder and President of CURE4WOLFRAM, INC. and employed by it. FU is an inventor of three patents related to the treatment of Wolfram syndrome, Soluble MANF in Pancreatic Beta Cell Disorders (US 9,891,231) and Treatment for Wolfram Syndrome and Other ER Stress Disorders (US 10,441,574 and US 10,695,324). The remaining authors declare that the research was conducted in the absence of any commercial or financial relationships that could be construed as a potential conflict of interest.

## Publisher’s Note

All claims expressed in this article are solely those of the authors and do not necessarily represent those of their affiliated organizations, or those of the publisher, the editors and the reviewers. Any product that may be evaluated in this article, or claim that may be made by its manufacturer, is not guaranteed or endorsed by the publisher.

## Abbreviations

MRI: magnetic resonance imaging
NfL: neurofilament light chain
ER: endoplasmic reticulum
ICV: intracranial volume
CSF: cerebrospinal fluid
MS: multiple sclerosis
AD: Alzheimer’s disease
WUSTL: Washington University in St. Louis
QC: quality control
WURS: Wolfram United Rating Scale
logMAR: Logarithm of the Minimum Angle of Resolution
UPSIT: University of Pennsylvania Smell Identification Test
MPRAGE: T1-weighted Magnetization-Prepared Rapid Gradient-Echo
eTIV: estimated total intracranial volume
ANCOVA: analysis of covariance
ANOVA: analysis of variance.
